# Subtype-specific role for Jagged1 in promoting or inhibiting breast tumor formation

**DOI:** 10.1038/s41389-025-00545-6

**Published:** 2025-01-31

**Authors:** Wen-Cheng Chung, Wei Wang, Lavanya Challagundla, Charles D. Moore, Sean E. Egan, Keli Xu

**Affiliations:** 1 Department of Cell and Molecular Biology, University of Mississippi Medical Center, Jackson, MS USA; 2 Cancer Center and Research Institute, University of Mississippi Medical Center, Jackson, MS USA; 3 Program in Cell Biology, The Peter Gilgan Center for Research and Learning, The Hospital for Sick Children, Toronto, ON Canada

**Keywords:** Breast cancer, Cell signalling

## Abstract

Notch signaling is altered in breast cancer. Recent studies highlighted both tumor-suppressive and oncogenic roles for Notch in this tissue. The function of Jagged1, the most highly expressed Notch ligand in the mammary gland, is not well defined. Here we report that deletion of Jagged1 in the mammary epithelium of virgin mice led to expansion of the mammary stem cell (MaSC) compartment and defective luminal differentiation associated with decreased expression of the progesterone receptor (PR). In contrast, deletion of Jagged1 in alveolar cells of pregnant mice had no effect on alveolar and lactogenic differentiation or post-lactational involution. Interestingly, deletion of Jagged1 promoted mouse mammary tumor formation from luminal cells but suppressed them from basal cells, associated with downregulation of Notch target genes Hey1 and Hey2, respectively. In agreement with mouse experiments, high expression of JAG1 and HEY1 are associated with better overall survival among patients with luminal tumors, whereas high expression of JAG1 and HEY2 are both associated with worse overall survival in basal subtype of human breast cancer. These results identified Jagged1 as an important regulator of mammary epithelial hierarchy and revealed differential roles of Jagged1-mediated Notch signaling in different subtypes of breast cancer arising from distinct cell types.

## Introduction

Breast cancer is a heterogeneous group of diseases that are thought to arise through transformation of several different mammary epithelial cell types. Based on gene expression profiling, molecular subtypes of human breast cancer have been defined. These include luminal A, luminal B, HER2-enriched, basal-like, claudin-low, and normal-like [1, 2]. The relationship between the normal mammary epithelial differentiation hierarchy and tumor subtypes have been postulated [3]. The Notch signaling pathway is an evolutionarily conserved signal transduction cascade that controls cell fate decisions, differentiation, and proliferation essential for both developmental morphogenesis and tissue homeostasis. Mammals have four variants of Notch receptors (Notch 1, 2, 3, and 4) and five Notch ligands (Dll 1, 3 and 4; Jagged 1 and 2). Notch receptors, ligands, as well as Notch downstream target Hes and Hey genes show dynamic expressions during postnatal mammary gland development, and Notch3 and Jagged1 represent the most highly expressed receptor and ligand, respectively [4]. Notch3 serves as a prominent receptor for the decision to adopt a luminal progenitor fate [5], and both tumor-suppressive and oncogenic roles for Notch3 have been identified in the mammary epithelium [6–9]. High-level Jagged1 expression was shown to predict poor outcomes in breast cancer and is associated with a basal-like phenotype [10–12], however, the function of Jagged1 in the mammary gland is not well defined.

Notch genes can function as oncogenes when hyperactivated or as tumor suppressors when deleted [13]. Indeed, this is now known to be true in mammary epithelium [14]. Divergent roles for Jagged1 have also been identified in breast cancers. Knockdown of JAG1 in luminal breast cancer MCF-7 cells resulted in increased tumorsphere growth and cancer stem cell activity [15]. To the contrary, JAG1 knockdown in triple-negative breast cancer SUM149 cells significantly restricted the growth of tumor organoids [16]. Lapatinib-mediated HER2 inhibition in HER2^+^ breast cancer cells upregulated JAG1 expression associated with enrichment of cancer stem cells [17]. These studies suggest a complex and context-specific function for Jagged1 in mammary epithelium and breast cancer. With knowledge of Jagged1 function(s) in both settings, subtype-specific Jagged1-based therapies could be imagined. This study aims to define roles of Jagged1 in normal mammary gland development as well as during tumor formation from different mammary epithelial cell types.

KRAS is one of the most frequently mutated genes in human malignancy. Whole genome sequencing found mutations in KRAS and its negative regulator NF1 in a subset of ER-negative breast tumors [18]. RAS pathway activation is thought to play an important role in breast cancer initiation and progression [19]. Indeed, human mammary basal cells and luminal progenitors can be transduced by oncogenic KRAS^G12D^ into serially transplantable invasive ductal carcinomas [20], and the activation of mutant KRAS in luminal cells induces preneoplastic lesions that progress to basal and claudin-low mammary tumors in mice [21]. Interestingly, oncogenic Ras activates Notch signaling and Notch is required for the maintenance of the neoplastic phenotype in Ras-transformed human cells [22]. In this study, we generated several mouse models for breast cancers by targeting mature luminal cells or basal/luminal progenitor cells with the combination of Kras^G12D^ and p53 deletion. By deleting *Jag1* in each model, we have identified subtype-specific cancer-suppressing and promoting functions for Jagged1.

## Results and discussion

### MMTV-Cre mediated deletion of Jagged1 causes accumulation of mammary stem cells and defective luminal differentiation in virgin mice

**Fig. 1:**
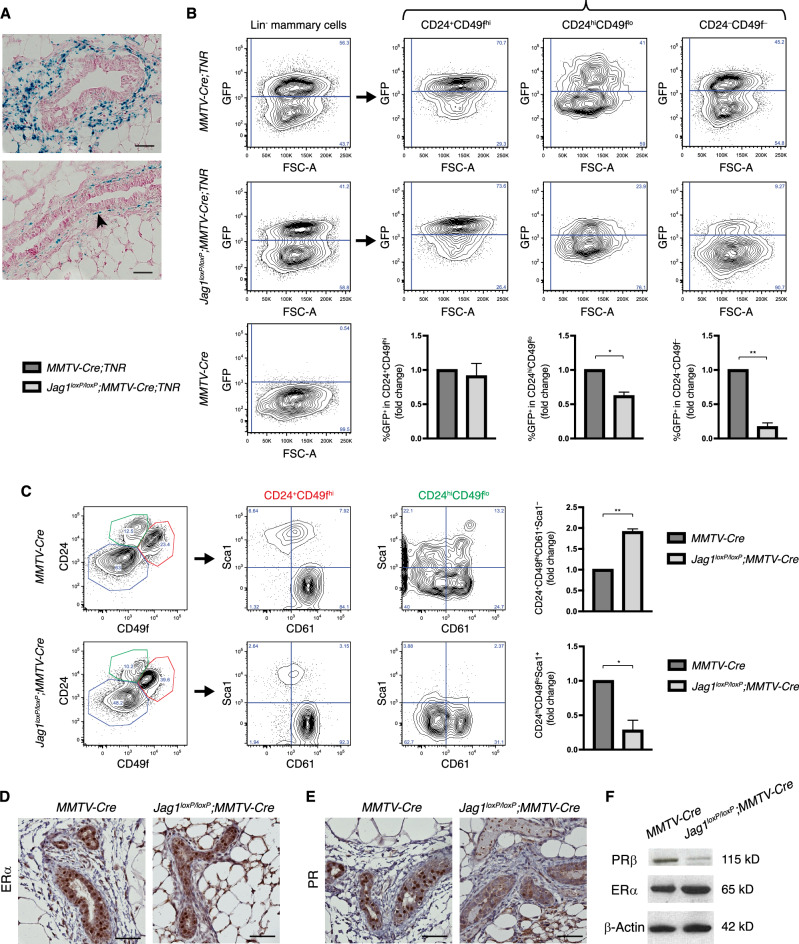
Mammary-specific deletion of Jagged1 in virgin mice resulted in reduced Notch activation in the luminal compartment, associated with expansion of MaSC and decreased luminal differentiation. **A** X-gal staining of mammary tissue sections from 6-week-old virgin *Jag1*^*β-Geo/+*^ mice showing TEB (upper) and mature duct (lower). **B** Representative flow cytometry analysis of lineage-depleted mammary cells isolated from virgin *MMTV-Cre;TNR* and *Jag1*^*loxP/loxP*^*;MMTV-Cre;TNR* mice at 7–10 weeks of age, with quantitation of GFP-positive cells in the CD24^+^CD49f^hi^ (basal), CD24^hi^CD49f^lo^ (luminal), and CD24^−^CD49f^−^ (stromal) compartments. *MMTV-Cre* mammary glands serve a negative control for GFP. **C** Flow cytometry analysis of lineage-depleted mammary cells from virgin *MMTV-Cre* and *Jag1*^*loxP/loxP*^*;MMTV-Cre* mice at 7–10 weeks of age and comparison of the CD24^+^CD49f^hi^CD61^+^Sca1^−^ (MaSC) and CD24^hi^CD49f^lo^Sca1^+^ (differentiated luminal cell) subpopulations between the two genotypes. **D**–**F** Immunostaining and Western blot analysis for ERα and PR in the mammary glands of *MMTV-Cre* and *Jag1*^*loxP/loxP*^*;MMTV-Cre* mice at 12 weeks of age. Scale bars: 50 μm. *p < 0.05; **p < 0.01 (Student’s t-test).

Jagged1 is expressed in stromal cells surrounding terminal end buds of the developing mammary gland as well as in basal cells of mature ducts from virgin mice (Fig. 1A) [23]. To understand its role in mammary gland development, we used MMTV-Cre line A [24] to delete the *Jagged1* gene in mammary epithelial cells of virgin mice. X-gal staining in the *Rosa*^*LSL-lacZ*^*;MMTV-Cre* mammary gland indicated that MMTV-Cre-mediated deletion occurred in almost all mammary epithelial cells but not in the stroma (Fig. S1A). *Jag1*^*loxp/loxp*^*;MMTV-Cre* mammary tissue showed a 60% decrease in Jagged1 mRNA level as compared to the *MMTV-Cre* control (Fig. S1B). Since Jagged1 expression was noted in mammary epithelium as well as the stroma, the remaining 40% Jagged1 mRNA in *Jag1*^*loxp/loxp*^*;MMTV-Cre* was likely in the stroma. We also determined the effects of Jagged1 deletion on the activation of Notch by crossing a Transgenic Notch Reporter (TNR) [25] into *Jag1*^*loxP/loxP*^*;MMTV-Cre* and *MMTV-Cre* mice. As *MMTV-Cre* mice show defective differentiation of mammary epithelium in late pregnancy [26, 27], we used this strain for comparison, and thereby controlled for any subtle defect unrelated to *Jagged1* loss in virgin animals. As expected, deletion of Jagged1 caused a reduction of the number of eGFP^+^ cells, indicative of reduced Notch reporter activation (Fig. 1B). Consistent with Jagged1 expression in basal cells, this decrease in Notch-reporter signaling was restricted to neighboring stromal (CD24^−^CD49f^−^) and luminal (CD24^hi^CD49f^lo^) compartment cells (Fig. 1B). Interestingly, *Jagged1*-mutant mammary glands showed expansion of the MaSC-enriched CD24^+^CD49f^hi^CD61^+^Sca1^−^ population, accompanied by a decrease in the number of terminally differentiated luminal cells (CD24^hi^CD49f^lo^Sca1^+^) [28] (Fig. 1C). The mutant gland exhibited normal expression of estrogen receptor-α (ERα), but significantly decreased progesterone receptor (PR) expression within the luminal compartment (Fig. 1D, E). This was confirmed by Western blot analysis (Fig. 1F). Despite defective luminal differentiation, Jagged1 deletion did not obviously affect ductal morphogenesis (Fig. S2). Taken together, mammary-specific deletion of Jagged1 in virgin mice resulted in decreased Notch activation in the luminal compartment, which was associated with defective luminal cell differentiation.

A major defect in *Jag1*^*loxP/loxP*^*;MMTV-Cre* mammary glands involved loss of PR expression. The mechanism on how Jagged1-mediated Notch signaling maintains PR expression is currently unknown. Activation of Notch signaling was shown to upregulate PR expression in decidual cells [29], and Notch signaling through RBPJk controls PR expression during decidualization [30], suggesting that canonical Notch signaling regulates PR expression. Our results revealed a nonredundant function for Jagged1 in the Notch regulation of PR in mammary epithelium. PR is downstream of ERα but also interacts with ERα to direct ERα chromatin binding and transcriptional activity. Co-expression of these two hormone receptors is associated with a good outcome for breast cancer patients [31]. Loss of Jagged1 in the luminal subtype may increase the propensity to ER^+^PR^−^, a status with higher risk of mortality than ER^+^PR^+^ [32, 33]. Of note, out of 5 cases harboring *JAG1* deep deletion or truncation in The Cancer Genome Atlas (TCGA) breast cancer dataset, three were ER^+^PR^−^ (60%), one ER^+^PR^+^ and one ER^−^PR^−^. Thus, Jagged1-mediated regulation of PR expression may have important implications in breast cancer, especially in ER^+^ tumors.

### Deletion of Jagged1 in alveolar cells during pregnancy and lactation does not affect lactogenic differentiation or post-lactational involution

Jagged1 continues to be expressed in myoepithelial cells at early stages of pregnancy (data not shown) but is upregulated in alveolar cells from mid to late pregnancy (Fig. S3A). Finally, alveolar cell expression of Jagged1 is downregulated at lactation and upregulated again at the start of post-lactational involution [7]. To define its function in alveolar cells of pregnant mammary glands, we deleted *Jagged1* in alveolar cells using WAP-Cre. Whole-mount preparation and histology of mammary glands at lactation day 10 showed normal alveolar and lactogenic differentiation in *Jag1*^*loxP/loxP*^*;WAP-Cre* mice as compared to *WAP-Cre* control (Fig. S3B). In addition, post-lactational involution as well as alveolar/lactogenic differentiation in subsequent pregnancy were normal in *Jag1*^*loxP/loxP*^*;WAP-Cre* mutants (Fig. S3B). Thus, Jagged1 expression in alveolar cells appears to be dispensable for the lactation and post-lactational involution.

### Jagged1 suppresses mammary tumor development from androgen-responsive luminal cells

**Fig. 2:**
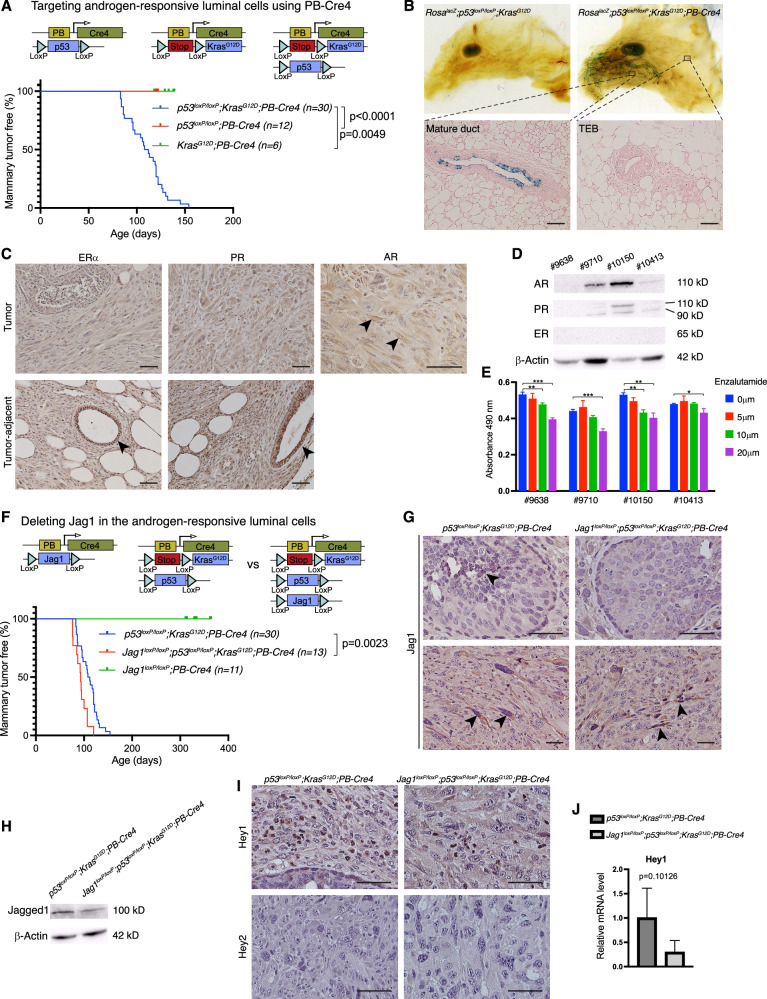
Deletion of Jagged1 promotes Kras^G12D^, p53^lof^-driven mammary tumor development from mature ductal luminal cells. **A** Schematic depiction of the targeting of androgen-responsive luminal cells using PB-Cre4, and Kaplan-Meier mammary tumor-free survival plots for the *p53*^*loxP/loxP*^*;Kras*^*G12D*^*;PB-Cre4*, *p53*^*loxP/loxP*^*;PB-Cre4*, and *Kras*^*G12D*^*;PB-Cre4* mice. **B** Whole-mount X-Gal staining of the mammary glands from *Rosa*^*lacZ*^*;p53*^*loxP/loxP*^*;Kras*^*G12D*^ and *Rosa*^*lacZ*^*;p53*^*loxP/loxP*^*;Kras*^*G12D*^*;PB-Cre4* mice at 6 weeks of age (upper) and sections of the *Rosa*^*lacZ*^*;p53*^*loxP/loxP*^*;Kras*^*G12D*^*;PB-Cre4* gland (lower). **C** Immunostaining for ERα, PR, and AR in the mammary tumors and adjacent non-tumor tissue from *p53*^*loxP/loxP*^*;Kras*^*G12D*^*;PB-Cre4* mice. **D** Western blot analysis for ER, PR, and AR in 4 independent breast cancer cell lines established from *p53*^*loxP/loxP*^*;Kras*^*G12D*^*;PB-Cre4* mice. **E** Growth of *p53*^*loxP/loxP*^*;Kras*^*G12D*^*;PB-Cre4* cell lines under treatment of 5, 10, and 20 μM Enzalutamide or vehicle control (DMSO). Relative numbers of viable cells were determined by MTS assay and presented as mean ± SD of the absorbance at 490 nm. **F** Schematic depiction of the deleting of Jag1 in androgen-responsive luminal cells, and Kaplan-Meier mammary tumor-free survival analysis in *Jag1*^*loxP/loxP*^*;PB-Cre4, p53*^*loxP/loxP*^*;Kras*^*G12D*^*;PB-Cre4*, and *Jag1*^*loxP/loxP*^*;p53*^*loxP/loxP*^*;Kras*^*G12D*^*;PB-Cre4* mice. **G** Anti-Jagged1 immunostaining in mammary tumors from *p53*^*loxP/loxP*^*;Kras*^*G12D*^*;PB-Cre4* and *Jag1*^*loxP/loxP*^*;p53*^*loxP/loxP*^*;Kras*^*G12D*^*;PB-Cre4* mice. **H** Western blot analysis for Jagged1 in *p53*^*loxP/loxP*^*;Kras*^*G12D*^*;PB-Cre4* and *Jag1*^*loxP/loxP*^*;p53*^*loxP/loxP*^*;Kras*^*G12D*^*;PB-Cre4* breast cancer cell lines. **I** Immunostaining for Hey1 and Hey2 in *p53*^*loxP/loxP*^*;Kras*^*G12D*^*;PB-Cre4* and *Jag1*^*loxP/loxP*^*;p53*^*loxP/loxP*^*;Kras*^*G12D*^*;PB-Cre4* mammary tumors. **J** Relative mRNA levels of *Hey1* in *p53*^*loxP/loxP*^*;Kras*^*G12D*^*;PB-Cre4* and *Jag1*^*loxP/loxP*^*;p53*^*loxP/loxP*^*;Kras*^*G12D*^*;PB-Cre4* tumors determined by quantitative RT-PCR. Scale bars: 50 μm. *p < 0.05; **p < 0.01; ***p < 0.001 (Student’s *t*-test).

To understand roles of Jagged1 in mammary tumor development, we generated mouse models targeting the luminal or basal cells of the mouse mammary epithelium. The PB-Cre4 transgenic line utilizes the *Pbsn* (probasin) gene promoter to direct Cre expression to prostatic epithelial cells [34]. We previously generated *p53*^*loxP/loxP*^*;Kras*^*G12D*^*;PB-Cre4* male mice as a model for metastatic prostate cancer [35]. Unexpectedly, female *p53*^*loxP/loxP*^*;Kras*^*G12D*^*;PB-Cre4* mice developed early onset mammary tumors but not at any other sites. Neither *p53*^*loxP/loxP*^*;PB-Cre4* nor *Kras*^*G12D*^*;PB-Cre4* mice formed tumors at the same age (Fig. 2A). To determine the mammary cell type in which PB-Cre4 is activated, we crossed *Rosa*^*LSL-lacZ*^ into *p53*^*loxP/loxP*^*;Kras*^*G12D*^*;PB-Cre4* and performed X-Gal staining. The *Rosa*^*LSL-lacZ*^*;p53*^*loxP/loxP*^*;Kras*^*G12D*^*;PB-Cre4* mammary gland showed robust staining in the ducts at 6 weeks of age, while mammary glands from Cre-minus control mice (*Rosa*^*LSL-lacZ*^*;p53*^*loxP/loxP*^*;Kras*^*G12D*^) were completely negative (Fig. 2B). Sectioning of the *Rosa*^*LSL-lacZ*^*;p53*^*loxP/loxP*^*;Kras*^*G12D*^*;PB-Cre4* gland revealed that luminal cells in the mature ducts, but not in the terminal end buds (TEB), showed lacZ activity (Fig. 2B). Thus, PB-Cre4 directed p53 deletion and Kras^G12D^ expression in mature luminal cells of the ductal system. Since *Pbsn* is regulated by activated androgen receptors (AR) [36], and AR signaling promotes a luminal phenotype in the mammary gland [37], it is conceivable that PB-Cre4 activity marks androgen-responsive luminal cells. *p53*^*loxP/loxP*^*;Kras*^*G12D*^*;PB-Cre4* tumors stained negative or very weak for ERα and PR, whereas ducal cells in adjacent non-tumor tissue stained positive for both receptors (Fig. 2C). Interestingly, these tumors stained positive for AR (Fig. 2C). Western blot analysis in four cell lines established from *p53*^*loxP/loxP*^*;Kras*^*G12D*^*;PB-Cre4* mammary tumors showed no expression of ER, weak expression of PR, and strong expression of AR in two cell lines (Fig. 2D). Enzalutamide is an AR inhibitor approved for the treatment of patients with metastatic castration-resistant prostate cancer and has been in clinical trial for the treatment of AR^+^ triple-negative breast cancer [38]. Indeed, pharmacological inhibition of AR by Enzalutamide had an inhibitory effect on the growth of these cells (Fig. 2E). Thus, *p53* deletion coupled with *Kras* activation in androgen-responsive luminal cells caused poorly differentiated ERα^−^ AR^+^ mammary tumors that are reminiscent of AR^+^ triple-negative breast cancers in humans [39].

Next, we crossed *Jag1*^*loxP/loxP*^ onto the *p53*^*loxP/loxP*^*;Kras*^*G12D*^*;PB-Cre4* model, where it significantly accelerated mammary tumor development, shortening the median tumor-free survival from 110 to 93 days (Fig. 2F). Of note, *Jagged1* deletion alone (*Jag1*^*loxP/loxP*^*;PB-Cre4*) did not result in tumor formation up to one year (Fig. 2F). As expected, Jagged1 immunostaining was seen in luminal cells from premalignant *p53*^*loxP/loxP*^*;Kras*^*G12D*^*;PB-Cre4* mice, but not in *Jag1*^*loxP/loxP*^*;p53*^*loxP/loxP*^*;Kras*^*G12D*^*;PB-Cre4* animals (Fig. 2G), and Western blot analysis in breast tumor cell lines established from these mice showed reduced Jagged1 protein level in the *Jag1* deletion mutant (Fig. 2H). Notably, Jagged1 expression was observed in spindle-shaped mesenchymal-like cells in tumors that formed in *Jag1*^*loxP/loxP*^*;p53*^*loxP/loxP*^*;Kras*^*G12D*^*;PB-Cre4* mice (Fig. 2G). Given the high-level Jagged1 expression in basal cells and fibroblasts (Fig. 1A) [40], Jagged1^+^ cells in these tumors are likely derived from basal cells or fibroblasts, neither of which appear to express Cre (in PB-Cre4 transgenics). Expression of Hey1, a canonical Notch target gene, showed decreased expression in the *Jag1*^*loxP/loxP*^*;p53*^*loxP/loxP*^*;Kras*^*G12D*^*;PB-Cre4* tumors vs. their Jagged1^+^ counterparts (from *p53*^*loxP/loxP*^*;Kras*^*G12D*^*;PB-Cre4* mice), whereas Hey2 was undetectable in the two tumor types (Fig. 2I,J). Since Hey1 was previously shown to repress AR-dependent gene expression [41], downregulation of Hey1 may lead to increased AR-dependent tumor cell growth. These results suggest that Jagged1 is tumor-suppressive in androgen-responsive luminal cells, and that Hey1 is a transcriptional target downstream of Jagged1-mediated Notch signaling in this context. Recent study suggests an association between AR expression and improved prognosis of ERα^+^ tumors. In contrast, AR expression is linked to worse prognosis in ERα^−^ breast tumors [42]. Jagged1-mediated Hey1 upregulation may therefore play a tumor suppressive role in ERα^−^ AR^+^ breast cancer via repression of AR downstream targets.

### Jagged1 promotes mammary tumor development from mammary stem and luminal progenitor cells

**Fig. 3:**
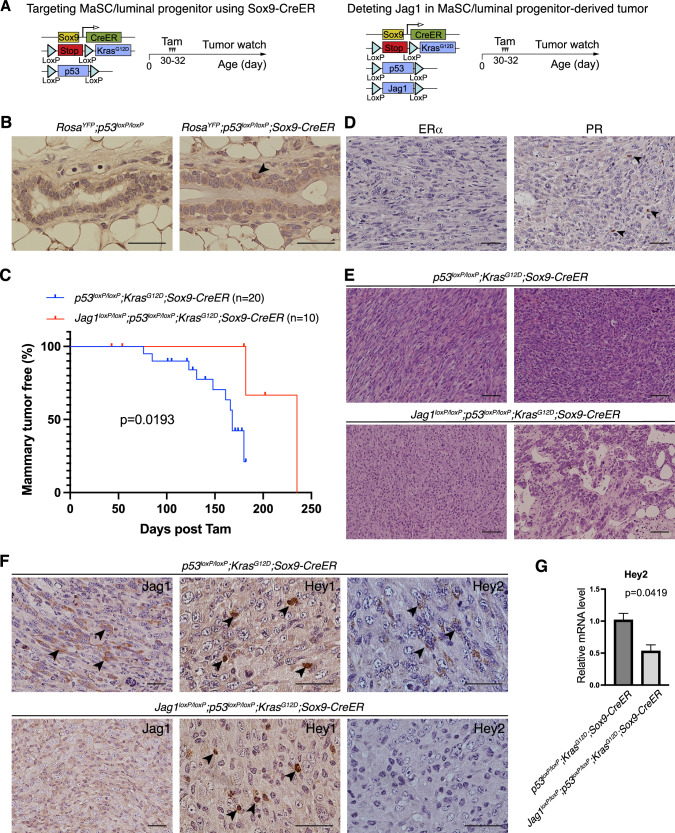
Deletion of Jagged1 inhibits Kras^G12D^, p53^lof^-driven mammary tumor development from mammary stem and luminal progenitor cells. **A** Schematic depiction of the targeting of MaSC/luminal progenitor using Sox9-CreER and deleting of Jagged1 in this model. **B** Anti-YFP staining in the mammary glands of 9-week-old *Rosa*^*YFP*^*;P53*^*loxP/loxP*^ and *Rosa*^*YFP*^*;P53*^*loxP/loxP*^*;Sox9-CreER* mice treated with tamoxifen at 5 weeks of age. **C** Kaplan-Meier mammary tumor-free survival plots for *p53*^*loxP/loxP*^*;Kras*^*G12D*^*;Sox9-CreER* and *Jag1*^*loxP/loxP*^*;p53*^*loxP/loxP*^*;Kras*^*G12D*^*;Sox9-CreER* mice. **D** Immunostaining for ERα and PR in mammary tumors from *p53*^*loxP/loxP*^*;Kras*^*G12D*^*;Sox9-CreER* mice. Arrows: rare PR^+^ tumor cells. **E** Representative histology of mammary tumors from *p53*^*loxP/loxP*^*;Kras*^*G12D*^*;Sox9-CreER* and *Jag1*^*loxP/loxP*^*;p53*^*loxP/loxP*^*;Kras*^*G12D*^*;Sox9-CreER* mice. Upper left: the spindle-shaped mesenchymal-like morphology; Lower right: partial glandular differentiation. **F** Immunostaining for Jagged1, Hey1, and Hey2 in mammary tumors from *p53*^*loxP/loxP*^*;Kras*^*G12D*^*;Sox9-CreER* and *Jag1*^*loxP/loxP*^*;p53*^*loxP/loxP*^*;Kras*^*G12D*^*;Sox9-CreER* mice. Arrows: positively stained cells. **G** Relative mRNA levels of *Hey2* in *p53*^*loxP/loxP*^*;Kras*^*G12D*^*;Sox9-CreER* and *Jag1*^*loxP/loxP*^*;p53*^*loxP/loxP*^*;Kras*^*G12D*^*;Sox9-CreER* mammary tumors determined by quantitative RT-PCR. Scale bars: 50 μm in B, D, F; 100 μm in E.

Previous studies showed that high-level JAG1 expression predict poor outcome [11], and JAG1 expression is associated with a basal breast cancer phenotype [12]. JAG1 expression in breast cancer cell is crucial for the growth in suspension and maintenance of mammospheres [43]. We sought to determine whether Jagged1 plays a tumor promoting role in MaSC and luminal progenitors, the putative cell-of-origin for basal-like breast cancer (Fig. 3A). Sox9-expressing cells in the mammary gland function as precursors for all mature lineages, as well as for luminal progenitors [44]. Indeed, lineage tracing using Rosa^LSL-YFP^ showed rare Sox9-expressing cells in the basal layer (Fig. 3B). Interestingly, Sox9-CreER mediated p53 deletion and Kras^G12D^ induction resulted in mammary tumor formation with a median onset at 168 days (post tamoxifen treatment) (Fig. 3C). The resulting tumors were poorly differentiated, ER-negative, had very few PR-positive cells, and contained spindle-shaped mesenchymal-like cells (Fig. 3D,E). To test for the role of Jagged1 in this mammary tumor type, we crossed *Jag1*^*loxP/loxP*^ onto the *p53*^*loxP/loxP*^*;Kras*^*G12D*^*;Sox9-CreER* model, and this resulted in a significant suppression of mammary tumor formation (Fig. 3C). Unlike mammary tumors in *p53*^*loxP/loxP*^*;Kras*^*G12D*^*;Sox9-CreER* mice, the sole tumor that formed in *Jag1*^*loxP/loxP*^*;p53*^*loxP/loxP*^*;Kras*^*G12D*^*;Sox9-CreER* mice contained very few spindle cells, instead, displayed glandular differentiation to some extent (Fig. 3E). As expected, mammary tumors from *p53*^*loxP/loxP*^*;Kras*^*G12D*^*;Sox9-CreER* but not *Jag1*^*loxP/loxP*^*;p53*^*loxP/loxP*^*;Kras*^*G12D*^*;Sox9-CreER* mice contained cells with strong Jagged1 immunostaining (Fig. 3F). Interestingly, while Hey1 immunostaining was similar in the two tumor types, the *Jag1*^*loxP/loxP*^*;p53*^*loxP/loxP*^*;Kras*^*G12D*^*;Sox9-CreER* tumor showed decreased expression of Hey2 protein and mRNA compared to *p53*^*loxP/loxP*^*;Kras*^*G12D*^*;Sox9-CreER* tumors (Fig. 3F,G). These results suggest that Jagged1-Notch-Hey2 signaling promotes development of basal-like mammary tumors from mammary stem cells and/or luminal progenitors.

Jagged1 deletion in luminal and basal cells led to decreased expression of Hey1 and Hey2, respectively. The reason for distinct Notch target genes downstream from Jagged1-mediated Notch activation is unclear. Further studies are warranted to determine whether a different Notch receptor is responding to Jagged1 in luminal vs. basal cells.

### High JAG1 expression is correlated to worse survival in patients with basal subtype of breast cancer but better survival with ER^+^PR^−^HER2^−^ tumors

**Fig. 4:**
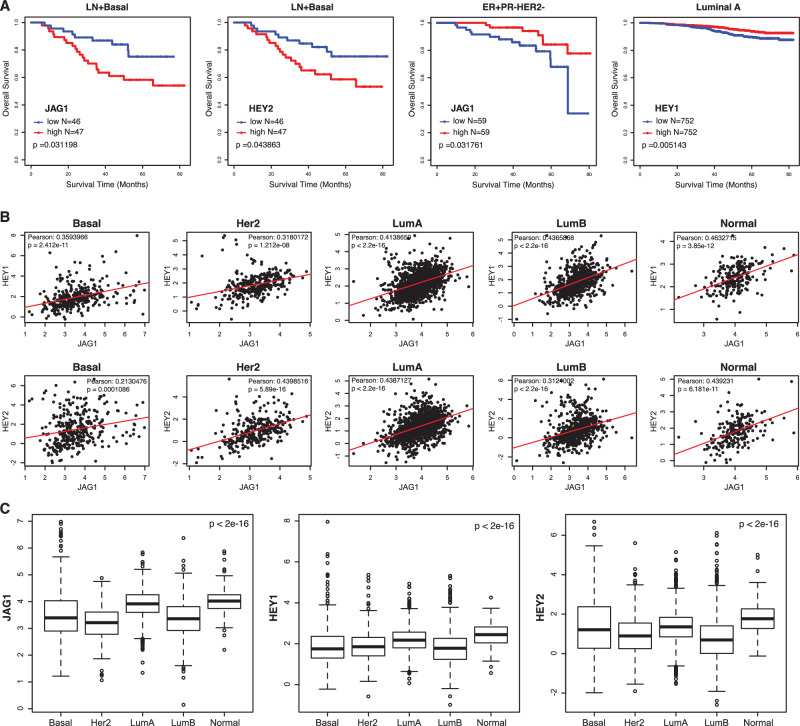
JAG1, HEY1, and HEY2 expressions and their correlation with overall survival in human breast cancer. **A** Overall survival rates in patients with lymph node positive basal subtype breast cancer related to expressions of JAG1 and HEY2, overall survival rates of patients with ER^+^PR^−^HER2^−^ breast tumors related to high or low expression of JAG1, and overall survival rates among patients with Luminal A subtype breast cancer related to HEY1 expression, using univariate Cox regression and Kaplan–Meier methods (dataset GSE96058). **B** Scatterplots for JAG1 and HEY1, JAG1 and HEY2 gene expressions in five molecular subtypes of human breast cancer (dataset GSE96058). **C** Boxplots for expression values of JAG1, HEY1, and HEY2 in five molecular subtypes of human breast cancer (dataset GSE96058). Basal: Basal-like. Her2: Her2-positive. LumA: Luminal A. LumB: Luminal B. Normal: Normal breast-like.

Deletion of Jagged1 had opposing effects on tumor development from different cell types of the mouse mammary gland (transformed by the same set of mutations). Crossing *Jag1*^*loxP/loxP*^ onto the *p53*^*loxP/loxP*^*;Kras*^*G12D*^*;Sox9-CreER* model caused a significant suppression of basal-like tumors arising from MaSC/luminal progenitors, suggesting a tumor-promoting role for Jagged1 in this tumor subtype. Indeed, high-level JAG1 expression is associated with poor survival in the basal subtype of human breast cancer (Fig. 4A). To the contrary, crossing *Jag1*^*loxP/loxP*^ onto the *p53*^*loxP/loxP*^*;Kras*^*G12D*^*;PB-Cre4* model accelerated mammary tumor development from mature luminal cells, revealing a tumor-suppressive function for Jagged1 in this cell type. Intriguingly, high JAG1 expression is associated with better survival among patients with ER^+^PR^−^HER2^−^ tumors (Fig. 4A), a subgroup belonging to the luminal B subtype [45]. Deletion of Jagged1 abolished PR expression in luminal cells of the mammary gland (Fig. 1E). Progesterone was shown to inhibit estrogen-fueled growth of ER^+^ cell line xenografts and primary ER^+^ breast tumor explants [31]. Thus, Jagged1 may suppress the ER^+^ luminal tumor by maintaining PR expression.

JAG1 expression is positively correlated with the expression of both HEY1 and HEY2 in all subtypes of human breast cancer (Fig. 4B). In parallel with JAG1, high HEY2 expression is associated with worse survival in basal subtype breast cancer (Fig. 4A), corroborating our finding in *p53*^*loxP/loxP*^*;Kras*^*G12D*^*;Sox9-CreER* mice, where deletion of Jagged1 suppressed basal tumor formation accompanied by decreased Hey2 expression. Interestingly, high expression of HEY1 is associated with better survival among patients with luminal A subtype (Fig. 4A). We also examined expression levels of the JAG1, HEY1 and HEY2 in different subtypes of human breast cancer. Notably, the luminal B subtype exhibits lower expressions of these genes compared to other subtypes, suggesting decreased Jagged1 activity in this subtype (Fig. 4C).

HEY2 was previously shown to play a role in maintaining breast cancer cell stemness and chemoresistance [46]. A recent single-cell analysis revealed that HEY2 controls expression of epithelial-to-mesenchymal (EMT) genes in metastatic breast cancer cells [47]. HEY1 was found to be upregulated following inhibition of ErbB-2, thus playing a role in resistance to trastuzumab in ErbB2-positive breast tumors [48]. HEY1 and HEY2 were co-expressed in a subset of ER^+^ breast tumors, and elevated expression of these two genes was significantly associated with distant metastasis and reduced overall survival [49]. Intriguingly, loss of Periostin in an ErbB2-overexpression tumor model resulted in apocrine-like tumors expressing low levels of Notch and Hey1 but increased expression of AR and AR target genes [50]. This is reminiscent of our finding in *p53*^*loxp/loxp*^*;Kras*^*G12D*^*;PB-Cre4* mice, where Hey1 may repress AR downstream target genes thereby inhibiting AR-driven tumor growth. Thus, the Notch target Hey family genes may play differential roles in breast cancers depending on the cell-of-origin and/or oncogenic signaling that fuel tumor development.

Collectively, results from our mouse experiments and patient datasets analysis support that Jagged1-mediated Notch signaling promotes basal-like breast cancer, a molecular subtype thought to arise from MaSC and/or luminal progenitors. To the contrary, Jagged1 may exert a tumor-suppressive role in the luminal compartment of the mammary epithelium.

## Materials and methods

### Mice

*Jag1*^*loxP*^ [51] and TNR [25] mice were kindly provided by Dr. Freddy Radtke and Dr. Nicholas Gaiano, respectively. *Jag1*^*β-geo*^ was generated previously [52]. Other strains including MMTV-Cre, PB-Cre4, Sox9-CreER, *p53*^*loxP*^, *Kras*^*LSL-G12D*^, *Rosa* ^*LSL-lacZ*^ and *Rosa*^*LSL-YFP*^ were received from the Jackson Laboratory and NCI Mouse Repository. Female *p53*^*loxP/loxP*^*;Kras*^*G12D*^*;Sox9-CreER* and *Jag1*^*loxP/loxP*^*;p53*^*loxP/loxP*^*;Kras*^*G12D*^*;Sox9-CreER* mice were administrated with tamoxifen (62.5 mg/Kg body weight) via i.p. injection on three consecutive days (one dose per day) at 4-5 weeks of age. No randomization was used. All animals were housed under standard condition and animal protocols were approved by Institutional Animal Care and Use Committees at the University of Mississippi Medical Center and the Hospital for Sick Children.

### Whole-mount and X-gal staining of mammary gland

Mouse inguinal mammary glands were harvested for whole-mount staining with hematoxylin. For X-gal staining, mammary glands were fixed in 1.0% formaldehyde supplemented with 0.02% Nonidet P-40 (in PBS) overnight, saturated with 30% sucrose, and embedded in Tissue-Tek O.C.T. compound (Sakura) for frozen sections, which were stained with X-gal for 4 to 12 h and counterstained with 0.5% eosin.

### Flow cytometry

Single cell suspensions were prepared from dissociated mouse mammary tissues using Collagenase/Hyaluronidase solution (StemCell Technologies) and lineage-depleted using EasySep mouse mammary stem cell enrichment kit (StemCell Technologies) according to the manufacturer’s instructions. Lin^−^ single cells were suspended in HBSS with calf serum and HEPES, and then stained with saturating concentrations of the following antibodies: eFluor 450 anti-mouse CD24 (eBioscience, 48-0242), PE-Cy5 anti-human CD49f (BD Pharmingen, 551129), PE-Cy7 anti-mouse Ly-6A/E (Sca-1) (eBioscience, 25-5981) and R-PE anti-mouse/rat CD61 (Invitrogen, MCD6104). Fluorescence was recorded using BD LSR-II flow cytometer and analyzed with FlowJo 9.1 (Treestar). Three independent flow cytometry experiments were performed using one control and one Jag1 mutant (littermates) for each experiment.

### Histology and immunohistochemistry

Mammary tissues were fixed in formalin, processed for paraffin-embedded blocks, then cut into 5 μm sections for histology and immunostaining. Tissue sections were rehydrated, followed by antigen retrieval with microwave, and stained with ERα (Santa Cruz, sc-542, 1:200), PR (Santa Cruz, sc-538, 1:200), AR (Santa Cruz, sc-816, 1:200), Jagged1 (Santa Cruz, sc-6011, 1:100), Hey1 (Proteintech, 19929-1-AP, 1:100), Hey2 (Proteintech, 10597-1-AP, 1:100), and YFP (ThermoFisher, A-11122, 1:200), using ImmunoCruz ABC Staining system (Santa Cruz) according to manufacturer’s instruction. At least two animals per genotype were used for each experiment and representative photomicrographs were acquired by investigators blinded to the genotype, using a Nikon Eclipse 80i microscope with NIS-Elements imaging software.

### Western blot analysis

Mouse mammary tissues were homogenized and lysed in RIPA buffer (Boston BioProducts) supplemented with protease inhibitor (Roche) and centrifuged for supernatants. Following quantification of total proteins using Pierce BCA Protein Assay Kit (Thermo Scientific), equivalent amounts from each sample were added with SDS loading buffer containing beta-mercaptoethanol and then heated at 95 ^o^C prior loading to Tris-Glycine-SDS-Polyacrylamide Gel. Antibodies for detecting specified proteins were ERα (Santa Cruz, sc-542), PR (Santa Cruz, sc-538), AR (Santa Cruz, sc-816), Jag1 (Santa Cruz, sc-390177), and β-Actin (Santa Cruz, sc-81178), all with 1:1000 dilution. At least two independent Western blots were performed using one control and one *Jag1* mutant (littermates) in each experiment.

### Drug treatment and MTS assay

Cell lines were established from mammary tumors in *p53*^*loxP/loxP*^*;Kras*^*G12D*^*;PB-Cre4* mice as previously described [53]. Cells were cultured in DMEM supplemented with 10% fetal bovine serum. For drug treatment, cells were incubated with 5, 10, and 20 μm of Enzalutamide (Selleckchem, S1250) dissolved in DMSO, or an equivalent amount of vehicle alone for 72 h. Cell viability was assessed using CellTiter 96 AQueous One Solution Cell Proliferation Assay (MTS) (Promega).

### Quantitative reverse transcription PCR

Total RNA was prepared using RNeasy Mini kit (Qiagen) and reverse-transcribed with iScript cDNA synthesis kit (Bio-rad). PCR was performed using QuantiTect SYBR Green PCR Kit (Qiagen) in BioRad CFX96 qPCR system, with primer sequences for *Hey1* and *Hey2* previously reported [54]. Results from triplicate PCR were normalized with the expression level of Gapdh.

### Human breast data sets analysis

Transcriptome-level gene expression dataset GSE96058 with available associated clinical information from human patients was downloaded from the GEO (https://www.ncbi.nlm.nih.gov/geo/) repository and analyzed in R ver. 4.3.0. Expression values for JAG1, HEY1 and HEY2 among different breast cancer subtypes was analyzed by one way ANOVA test and significance (*p*) values calculated by comparing expression means across all subtypes. For the survival analysis related to JAG1, HEY1 and HEY2 expression, we used the R package survival ver. 3.7.0 to perform univariate Cox regression analysis and Kaplan Meier survival curves with median as cutoff. Pearson correlations were calculated for the coexpression of the above mentioned genes and two sided *p* values reported.

### Statistics

Statistical analyses were performed using Prism version 10.1.1 (GraphPad Software). Data were presented as the mean with standard deviation (SD) and compared using two-tailed Student’s t-test. Mammary tumor-free survival was calculated by the Kaplan-Meier method and compared by nonparametric log-rank test. *P*-values < 0.05 were considered to indicate statistically significant differences for all of our analyses.

## Supplementary information

Supplemental Figure Legends

Supplemental Figure 1

Supplemental Figure 2

Supplemental Figure 3

## Data Availability

All relevant data is available from the authors upon request.
