# Supplementary material for: Subtype-specific role for Jagged1 in promoting or inhibiting breast tumor formation
Source: Oncogenesis. 2025 Jan 31;14(1):2. doi: 10.1038/s41389-025-00545-6 (PMC11785972; doi:10.1038/s41389-025-00545-6)
Supplement: Supplementary file 1 — Supplemental Figure Legends [file 41389_2025_545_MOESM1_ESM.docx]

**Supplemental figure legends**

**Fig. S1: MMTV-Cre-mediated deletion of Jagged1 in the mammary epithelial cells.**

**A** X-Gal staining of the mammary gland sections from *R26^LacZ^;MMTV-Cre* mice at 6 weeks old (virgin) and at pregnancy day 17.5. Scale bars: 50 μm. **B** Relative mRNA levels of *Jag1* in *MMTV-Cre* and *Jag1^loxP/loxP^;MMTV-Cre* mammary tissues determined by quantitative RT-PCR. P=0.0014 (Student's t-test).

**Fig. S2: Mice with mammary-specific deletion of Jagged1 showed normal mammary ductal morphogenesis.**

Representative whole-mount mammary glands from *MMTV-Cre* and *Jag1^loxP/loxP^;MMTV-Cre* virgin mice at 6 weeks and 16 months of age. Scale bars: 3 mm.

**Fig. S3: Jagged1 is expressed in the mammary gland during pregnancy but deletion of Jagged1 had no effect on lactation and post-lactational involution.**

**A** X-Gal staining of mammary tissue from *Jag1^β-geo/+^* mice at pregnancy day14.5 and 17.5. Panels at right are high magnification images of the squared areas in left panels. **B** Representative whole-mount preparations and histology of mammary glands from *WAP-Cre* and *Jag1^loxP/loxP^;WAP-Cre* mice at lactation day 10, involution day 6, and lactation day 5 following the second pregnancy. Scale bars: 50 μm in **A** and 100 μm in **B**.
