# Supplementary figures and images for: Subtype-specific role for Jagged1 in promoting or inhibiting breast tumor formation
Source: Oncogenesis. 2025 Jan 31;14(1):2. doi: 10.1038/s41389-025-00545-6 (PMC11785972; doi:10.1038/s41389-025-00545-6)

**A** *Rosa<sup>LacZ</sup>;MMTV-Cre*

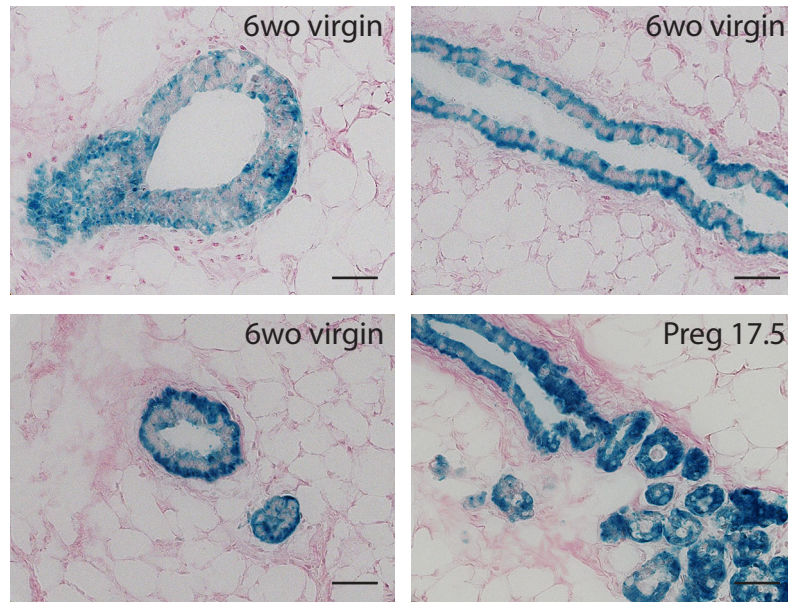

**B**

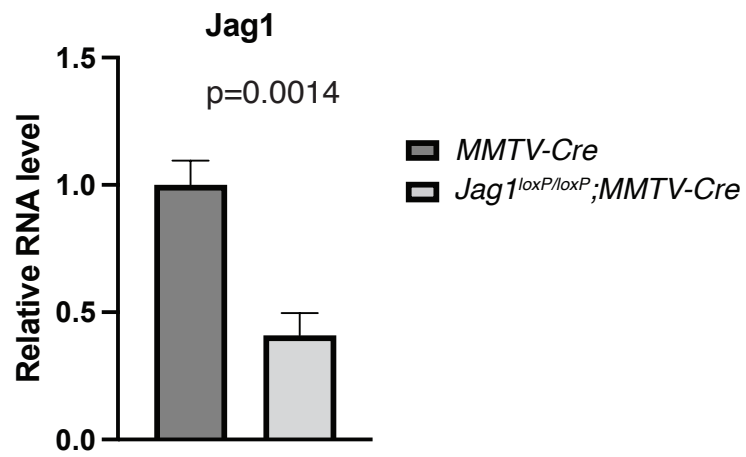

Supplement: Supplementary file 2 — Supplemental Figure 1 [file 41389_2025_545_MOESM2_ESM.pdf]

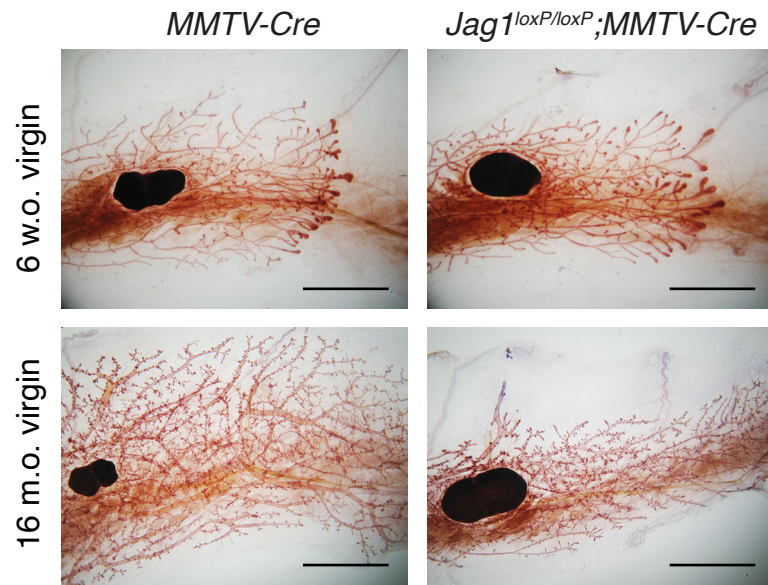

Supplement: Supplementary file 3 — Supplemental Figure 2 [file 41389_2025_545_MOESM3_ESM.pdf]

**A**

Preg 14.5

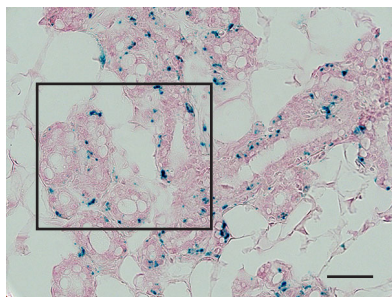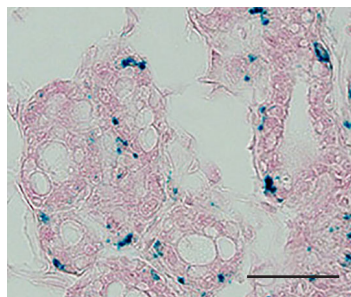

Preg 17.5

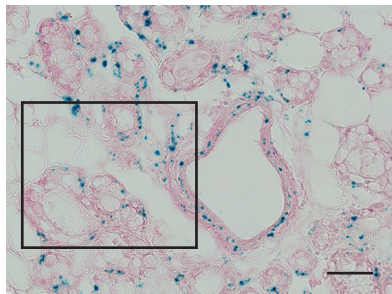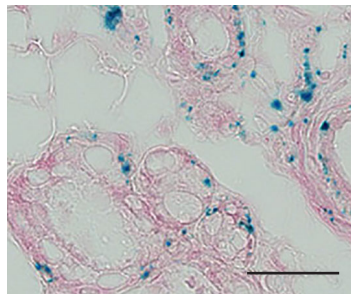**B***Jag1<sup>loxP/loxP</sup>**Jag1<sup>loxP/loxP</sup>;WAP-Cre*

Lac 10

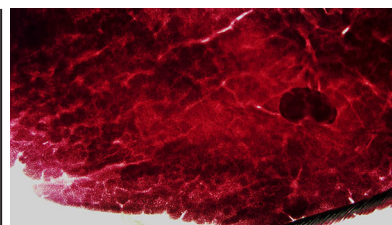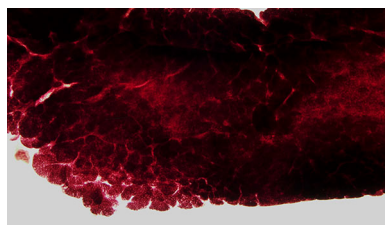

Inv 6

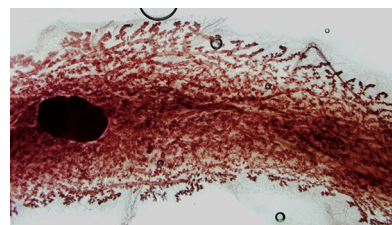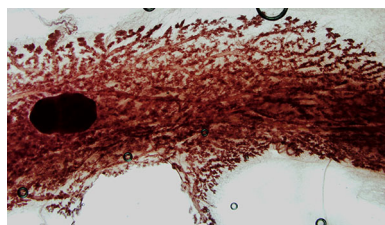Lac 5 (2<sup>nd</sup> preg)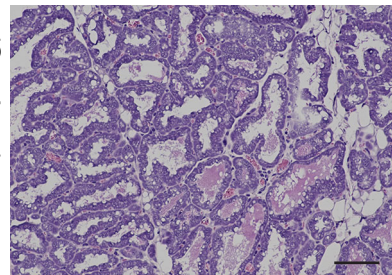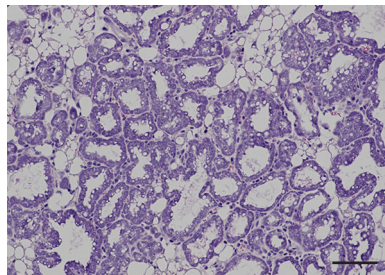

Supplement: Supplementary file 4 — Supplemental Figure 3 [file 41389_2025_545_MOESM4_ESM.pdf]
